# Plastic waste management, a matter for the ‘community’

**DOI:** 10.1111/1751-7915.13328

**Published:** 2018-11-08

**Authors:** Oliver Drzyzga, Auxiliadora Prieto

**Affiliations:** ^1^ Polymer Biotechnology Laboratory Microbial and Plant Biotechnology Department Centre for Biological Research (CIB‐CSIC) Madrid Spain; ^2^ Interdisciplinary Platform for Sustainable Plastics towards a Circular Economy‐Spanish National Research Council (SusPlast‐CSIC) Madrid Spain

Worldwide plastic production has surged over the past 50 years. In 2016, it reached 335 million tonnes per annum, with Europe alone producing 60 million tonnes. Over the next 20 years, it is expected to double. Plastic packaging is the most important product (26% of the total volume of all plastics used), although it has a short life compared to plastics used in, for example, the construction and car industries. Plastic producers and transformers are keen to highlight the benefits derived from plastic packaging; not only does it deliver direct economic profits, but it also helps prevent food waste and contamination. Further, by lessening the weight of packaging, it can reduce the fuel used in the transport of goods. This is certainly important, but even if these plastics are re‐used, they inevitably become waste at some point. If we are to close the loop of the circular economy, this waste needs to be seen as a resource to be plugged back into the life cycle of plastics (PlasticsEurope, 2018).

Unfortunately, a very large quantity of plastic waste leaks into the environment causing significant economic and ecological damage. For example, some 5–13 million tonnes of plastic (1.5–4% of global plastic production) end up in the ocean every year (Geyer *et al*., 2017). Educational campaigns are now focusing on the idea of citizens understanding themselves as members of a global community that can reduce the demand for plastic. However, according to all current expert reports, if the advantages of plastics are to be enjoyed in full, we also need to promote the most sustainable waste management alternatives, encourage recycling, use energy recovery as a complementary option and restrict the dumping in landfills of all recoverable plastic waste.

Of the 25.8 million tonnes of plastic waste generated in Europe every year, under 30% is collected for recycling; 31% ends up in landfills and 39% is incinerated. Within this context, the European Strategy for Plastics in a Circular Economy, adopted on 16 January 2018, aims to transform the way plastic products are designed, produced, used and recycled in the EU. The most challenging goals laid out include those of ensuring that, by 2030, all plastic packaging in the EU should be reusable or recyclable in a cost‐effective manner, and that more than half of all plastic waste generated in Europe be recycled (European Commission, 2018).

Mechanical recycling is currently the most common method used to recycle plastic waste (Ragaert *et al*., 2017); the term covers its collection, sorting, washing and grinding. The actual procedures followed depend on the origin and composition of the waste. For example, postindustrial (PI) wastes are usually clean, have no organic residues and are of known composition. In contrast, postconsumer wastes (PC) are often mixed polymer wastes with many organic and inorganic impurities – a huge challenge for recycling. Four polymers – high‐density polyethylene (HDPE), low‐density polyethylene (LDPE), polypropylene (PP) and polyethylene terephthalate (PET) – dominate the plastic waste derived from PC packaging. PC is by far the biggest fraction of plastic packaging waste and the most difficult to deal with. However, some common challenges arise when mechanically recycling both PI and PC. The main issue is the fact that, under certain heat, oxidation, radiation, hydrolysis and mechanical shear conditions, polymers of both types degrade in an uncontrolled manner. Indeed, the degradation that occurs during a PC's long‐term exposure to such factors can be very significant. An additional challenge for the recycling of mixed plastic waste is the differences in the melting points and processing temperatures of the different polymers involved.

Drawbacks like these have led to a growing interest in chemical and biotechnological recycling technologies. Chemical recycling involves transforming a plastic's polymers into its smaller oligomers or monomers, which can then be converted into chemicals, fuels or virgin plastics. Chemical recycling routes are generally divided into thermochemical or catalytic conversion processes, but can involve their combination. Well‐known processes include gasification, pyrolysis and catalysed cracking (Ragaert *et al*., 2017). Pyrolysis is an attractive technology for plastics that are currently incinerated or dumped in landfills due to intrinsic difficulties in mechanical or chemical recycling. Such is the case, for example, of mixed multilayer films, which are harder to recycle than the metal, paper and glass containers they have replaced. Against this background, some sustainable initiatives have been started. For instance, hybrid bio‐based high oxygen/water barriers and active coatings are being developed for use in monolayer bio‐based food packaging (films and trays) in a joint industrial and academic initiative. This could provide an alternative to current metalized packaging. It aims to avoid the use of non‐renewable materials in multilayer structures that currently require complex and expensive recycling steps (www.refucoat.eu). These hybrids, involve bio‐based polymers such as polyhydroxyalkanoates (PHA) – cost competitive polymers with good water barrier properties – and polyglycolic acid (PGA), which has excellent water barrier properties and is one of the most promising novel barrier polymers commercially available. Other biotechnological alternatives in the pipeline include the use of biocatalysts (bacterial cells and enzymes) for both plastic production and waste management.

Polyethylene and PET products are traditionally considered non‐biodegradable, but there are indications that they can be degraded, transformed and metabolized by microbes (Alshehrei, 2017). Several enzymes have been identified that can hydrolyse ester‐containing PET and other polyester plastics such as polyurethane (PU; Wierckx *et al*., 2018). The degradability of these plastics, however, greatly depends on the type of molecular bonds present in their polymers. Plastics containing hydrolysable bonds in their backbones, such as ester or urethane bonds, can be depolymerized by microbial polyester hydrolases, lipases, proteases and other enzymes (Wierckx *et al*., 2018). By screening natural microbial communities exposed to PET in the environment, Yoshida *et al*. (2016) isolated a novel bacterium (*Ideonella sakaiensis* strain 201‐F6) that can use PET as its major energy and carbon source. PEs containing only carbon–carbon bonds in their backbones are obviously recalcitrant to biological attack and are rarely reported to be degraded (Wei and Zimmermann, 2017). However, a combination of abiotic (e.g. UV light and high temperatures) and biotic action can lead to their breakdown in the environment. Amongst the biotic factors, and in addition to the above‐mentioned enzymes, several oxidoreductases have been shown to degrade PE (Lucas *et al*., 2008). The resulting monomers can be used to provide a carbon feedstock for other microorganisms and therefore used to produce new products with added value.

Engineering enzymes for plastic degradation are emerging as a new field of study. Austin *et al*. (2018) characterized the three‐dimensional structure of a newly discovered plastic‐degrading aromatic polyesterase that can digest highly crystalline PET (PETase). In their study, they engineered this enzyme for improved PET degradation capacity and showed that it can also degrade polyethylene‐2,5‐furandicarboxylate (PEF, an important PET replacement), opening up new opportunities for bio‐based plastic recycling. Further engineering to increase the performance of PETase is possible and realistic, and underlines the need for further research into structure/activity relationships that might be of interest in the biodegradation of synthetic polyesters.

Microbial populations and communities (both natural and designed) may become key in plastic degradation by being able to use feedstock and building block compounds (e.g. synthesis gas, carbon‐containing monomers and oligomers) resulting from the thermochemical and chemical recycling of plastic. These could be used to produce *de novo* products by fermentation. Natural or designed microbial communities might also be used for the biodegradation of petroleum‐based plastic waste, with a balanced set of enzymes attacking the carbon backbones under favourable abiotic conditions (e.g. at controlled industrial composting facilities; Bhardwaj *et al*., 2012). This was recently demonstrated in a marine microcosm by Syranidou *et al*. (2017), who examined the potential of the bacterially mediated degradation of naturally weathered polyethylene (PE) films. Using an indigenous marine community alone or bio‐augmented with strains able to use linear low‐density polyethylene (LLDPE) as their sole carbon source for a few months, active biofilms were established on PE, leading to the establishment of efficient PE‐degrading microbial networks.

Designed bacterial communities (perhaps together with aggressive fungal strains) might therefore have a future in the degradation of waste plastic. Studies involving high‐throughput sequencing techniques to characterize the microbial communities on plastics have focused on their composition. For example, Skariyachan *et al*. (2016) tried to formulate novel microbial communities isolated from plastic garbage processing areas to demonstrate the possibility of eco‐friendly enhanced degradation of low‐density polyethylene (LDPE) strips and pellets. The LDPE‐degrading bacteria were screened and microbiologically characterized, and weight reductions of 81% (±4) and 38% (±3) were, respectively, recorded for LDPE strips and LDPE pellets over an incubation period of 120 days. This study (amongst others) suggests that scaling‐up these strategies might afford an interesting alternative for the management or recycling of waste LDPE and similar types of plastic garbage.

It has also been shown that several fungi have the potential to degrade PE in aquatic and soil environments. It was also recently shown that a marine fungus, *Zalerion maritimum*, can degrade PE (Paço *et al*., 2017). To maximize the chance of identifying plastic‐degrading microorganisms in the environment, the fungal community on plastic debris should be studied. Recently, Munir *et al*. (2018) isolated and identified the LDPE‐degrading fungi *Trichoderma viride* and *Aspergillus nomius* in a landfill soil in Medan (Indonesia) and showed them to degrade LDPE film over a 45‐day incubation period.

In conclusion, it may be possible to design efficient microbial communities able to degrade plastic waste – even those types currently recalcitrant to biologically driven breakdown. The integration of mechanical, chemical, thermochemical and biotechnological recycling techniques with microbial, fungal and even protist biological activity allowed to proceed under controlled and contained conditions, may perhaps be the key to attaining the goal of a circular economy in this sector.

## Conflict of interest

None declared.
